# COVID-19-related stress in Italy: a comparison between patients with mental disorders and the general population

**DOI:** 10.1192/j.eurpsy.2023.1237

**Published:** 2023-07-19

**Authors:** M. N. Modesti, S. Mimun, A. Bruzzese, V. Carola, G. Nicolais, C. Lai, A. Del Casale

**Affiliations:** 1 Faculty of Medicine and Psychology; 2 Department of Dynamic and Clinical Psychology and Health Studies, Faculty of Medicine and Psychology, Sapienza University of Rome, Rome, Italy

## Abstract

**Introduction:**

Following the surge of the COVID-19 pandemic, some people have been experiencing severe mental health consequences related to pandemic stress, fear of contagion, lockdown, and measures to avoid contagion and virus spread. These aspects contributed to an increase in anxious-depressive symptoms in the general population (Asmundson *et al.* J Anx Dis 2020; 70 102196).

**Objectives:**

The study aims at verifying the hypothesis that Italian patients with a diagnosis of a mental disorder showed more severe depressive, anxiety and stress-related symptoms compared to the general Italian population in the context of the current pandemic.

**Methods:**

Nine hundred sixty-one volunteer subjects (542 females, 415 males; mean age 39.42, SD = 14.5) completed the Covid-Stress-Scale (CSS) (Taylor *et al.* J Anx Dis 2020; 72 102232) and the Depression-Anxiety-Stress Scales-21 (DASS-21) (Bottesi *et al*. Compr Psych 2015; 60 170-81) through a self-report survey. Participants have been assessed for between-group differences through the chi-square test for categoric variables and one-way ANOVA for continuous variables.

**Results:**

One hundred and thirty subjects (13.53% of the whole sample) reported a diagnosis of a mental disorder for which they received medications. Among these subjects, 47.8% reported a diagnosis of anxiety disorder, 29% major depressive disorder, 2.7% bipolar disorder, and 20.4% other mental disorders. Among patients, there was a prevalence of females (chi-square = 15.84; p < 0.001), more severe depressive (F = 34.25; p < 0.001), anxiety (F = 46.15; p < 0.001), and stress-related symptoms (F = 39.38; p < 0.001) at the DASS-21 scale. The patient group also showed a tendency to more severe traumatic stress related to the pandemic (F = 3.64; p = 0.057) at factor IV of the CSS, without significant differences in the other factors of the CSS.

**Conclusions:**

The hypothesis is partially confirmed, considering that patients showed more severe depressive, anxiety and stress-related symptoms and a tendency to more severe pandemic traumatic stress. Nevertheless, in all other pandemic-related symptoms we analyzed (i.e., xenophobia, increase of medical assessments, fear of contagion), there were no differences between the group of patients and the general population. In this sense, in the current scenario in Italy, symptoms directly related to pandemic stress are almost the same in both the general population and patients with mental disorders.

**Disclosure of Interest:**

None Declared

